# Poloxamer dilution as an on-demand alternative to agar dilution-based antimicrobial susceptibility testing

**DOI:** 10.1128/jcm.01822-25

**Published:** 2026-03-05

**Authors:** Matthew T. J. Uy, Andrea Kirmaier, Lindsey M. Rudtner, Aidan Pine, James E. Kirby

**Affiliations:** 1 Beth Israel Deaconess Medical Center 1859 , Boston, Massachusetts, USA; 2 Northeastern University 1848, Boston, Massachusetts, USA; 3 Harvard Medical School 1811 , Boston, Massachusetts, USA; Children's Hospital Los Angeles, Los Angeles, California, USA

**Keywords:** Poloxamer 407, Pluronic F-127, agar dilution, antimicrobial susceptibility testing, fosfomycin, Mueller–Hinton medium, gram-negative bacteria, *Enterobacterales*, *Pseudomonas aeruginosa*, clinical microbiology laboratory

## Abstract

Agar dilution is a reference susceptibility testing method uniquely or preferentially recommended for certain antimicrobials. However, the effort required to pour individual agar plates spanning a doubling dilution range precludes its practical implementation in hospital clinical laboratories. Here, we describe an on-demand replacement for agar dilution, specifically substituting Poloxamer 407 (also known as Pluronic F-127) for Bacto agar as the solidifying agent. Notably, 20% Poloxamer 407 solutions (e.g., with Mueller–Hinton broth) remain liquid at refrigerated temperatures but solidify upon warming, enabling facile setup of poloxamer dilution testing in Petri dish or microwell format. For fosfomycin susceptibility testing, poloxamer dilution and reference agar dilution showed excellent categorical agreement (CA) and essential agreement (EA) for *Escherichia coli* (100% and 87%, respectively, *n* = 31). For other *Enterobacterales*, excluding *Klebsiella* spp., CA and EA were both 82% (*n* = 17, respectively). For *Pseudomonas aeruginosa*, CA and EA were 60% and 100% (*n* = 10), respectively, with the lower CA reflecting the large number of strains tested with minimal inhibitory concentrations near categorical breakpoints. There were no very major errors, while major errors were only observed for *Klebsiella* spp. Additionally, poloxamer dilution substantially reduced the number of skipped dilutions sixfold for *E. coli* (*P* < 0.0001) and inhibited swarming of *Proteus* spp. We conclude that poloxamer dilution and agar dilution, an imperfect gold standard, have essentially equivalent practical performance and that poloxamer dilution can therefore serve as an on-demand alternative testing methodology in clinical laboratories for fosfomycin testing of gram-negative pathogens. A broader exploration of poloxamer dilution’s utility is thus warranted.

**IMPORTANCE:**

Accurate antibiotic susceptibility testing is essential for guiding treatment of bacterial infections. For the antibiotic fosfomycin, used to treat *Escherichia coli* urinary tract infections, the most reliable testing method requires solid media prepared by hand for each antibiotic concentration, which is too time-consuming for most clinical laboratories to perform. Our study shows that replacing agar with an alternative temperature-sensitive gelling agent called poloxamer enables laboratories to prepare solid test plates rapidly without special equipment. This approach, which is essentially identical to traditional agar dilution, provides a practical means for performing reference-quality minimal inhibitory concentration (MIC) testing near the point of patient care, as demonstrated for fosfomycin, for which current FDA-cleared methods do not provide MIC data. This strategy may also be applicable to other drugs for which agar dilution is the preferred testing method, supporting expedited testing to inform treatment decisions for bacterial infections.

## INTRODUCTION

Antimicrobial resistance is emerging as a significant concern for the treatment of bacterial infections. To identify appropriate therapies for bacterial infections, clinical laboratories must therefore routinely conduct antimicrobial susceptibility testing (AST) on patient isolates. Manually prepared broth or agar antimicrobial susceptibility testing panels are considered the reference gold standard for the determination of minimal inhibitory concentration (MIC) values (1). In broth microdilution, the MIC is defined as the lowest concentration of an antimicrobial that visibly inhibits bacterial growth after a specified incubation period (1–3). For agar dilution, the MIC corresponds to the lowest agar plate concentration, in a twofold dilution series, that shows no visible growth of the test isolate. MICs are categorized as susceptible, susceptible dose-dependent, intermediate, or resistant based on CLSI, FDA, or EUCAST standards to guide antibiotic therapy selection.

Existing reference AST methods are labor intensive and technically complex, precluding their use in most hospital clinical laboratories near the site of patient care. Therefore, standard hospital-based clinical laboratory AST is generally performed using automated commercial platforms based on variants of broth dilution testing or alternatively by disk diffusion or gradient diffusion methods (4). However, these methods may not be appropriate for testing diffusion-limited drugs.

For certain drug–organism combinations, agar dilution is either the only or the preferred CLSI testing method (1–3). A prominent example is fosfomycin, an antimicrobial commonly prescribed for treatment of uncomplicated urinary tract infections (uUTIs) for which agar dilution serves as the reference method. Broth microdilution and gradient diffusion methods exhibit suboptimal accuracy and reproducibility. However, agar dilution itself is associated with a high frequency of skipped dilutions (the equivalent of “skipped wells” in broth microdilution testing) (5, 6), which precludes the reliable use of single-concentration agar breakpoint screening plates, as such irregularities could yield unrecognized errors.

CLSI fosfomycin disk diffusion breakpoints are available for *Escherichia coli* and *Enterococcus faecalis*, but not for other organisms, because the drug’s FDA approval is limited to treatment of uUTI caused by these pathogens. Moreover, disk diffusion-based methods correlate poorly with agar dilution testing outside these organism groups (7). Nevertheless, because fosfomycin may be the only oral agent with potential activity against certain gram-negative urinary pathogens, clinical laboratories are frequently asked to extrapolate *E. coli* disk diffusion breakpoints to other organisms.

It would be ideal if accurate fosfomycin MIC results for *E. coli* and other organisms could be generated near the site of patient care. This need arises because reference laboratory testing results are necessarily delayed, and reference laboratories also generally do not perform MIC testing in the absence of species CLSI breakpoints. While CLSI interpretive breakpoints for fosfomycin are limited, determination of reference MIC values remains clinically informative and is commonly used to support expert consultation, pharmacokinetic (PK)–pharmacodynamic (PD) assessment, and off-label therapy decisions for multidrug-resistant gram-negative urinary tract infections, consistent with CLSI guidance and EUCAST principles regarding MIC distributions and PK/PD-based interpretation in the absence of clinical breakpoints (3, 8, 9).

We have identified a potential way to address this issue based on technology developed in prior work. Previously, our group developed a rapid microscopy-based antimicrobial susceptibility testing platform that met FDA accuracy criteria within 4 hours of organism inoculation (10). As part of this work, we found that organisms could reliably grow on an optically clear Mueller–Hinton medium solidified with Poloxamer 407, which fully supported the growth of a wide range of gram-negative and gram-positive pathogens. Poloxamer 407 possesses a unique and beneficial physical property in solution: it remains liquid when refrigerated but transitions to a solid gel at higher temperatures (11–13). We therefore hypothesized that a cold, sterile liquid poloxamer stock solution could substitute for standard Bacto agar as the solidification reagent, providing a flexible and readily implemented alternative to agar dilution.

The potential advantages of such a solidification agent are numerous. Poloxamer media aliquots can be sterilized by autoclaving and stored at 4°C until use, then gently mixed with doubling dilutions of antibiotics, solidified at room temperature in less than 30 minutes, and subsequently inoculated and read using standard agar dilution protocols. This approach could therefore enable an agar dilution-equivalent method that can be prepared easily and on demand, eliminating the need for the repetitive, time-consuming process of autoclaving media, holding molten agar at a narrow temperature range, and manually incorporating doubling dilutions of antibiotics.

We therefore sought to validate poloxamer dilution technology compared with reference agar dilution testing using fosfomycin antimicrobial susceptibility testing as a representative use case.

## MATERIALS AND METHODS

### Bacterial strains

Fosfomycin-susceptible *E. coli* ATCC 25922 and *E. faecalis* ATCC 29212 were obtained from the ATCC. *E. coli* AR-346 and AR-549 are fosfomycin-resistant *fosA* isolates obtained from the FDA-CDC AR Bank collection (14). An additional 78 de-identified, colony-purified clinical isolates were retrieved from the Beth Israel Deaconess Medical Center Clinical Microbiology Laboratory, as listed in Table S1, under an IRB-approved protocol. These comprised *Escherichia coli* (*n* = 31), *Klebsiella* spp. (*n* = 20), *Pseudomonas aeruginosa* (*n* = 10), *Proteus* spp. (*n* = 8), *Citrobacter* spp. (*n* = 4), *Enterobacter cloacae* complex (*n* = 2), and one isolate each of *Providencia rettgeri*, *Salmonella* spp., and *Morganella morganii*.

### Media preparation

The following reagents were used for media preparation: Pluronic F-127 (Sigma-Aldrich; P2443-250G, lot BCBV1572), non-cation-adjusted Mueller–Hinton broth (BD Difco; 275730, lot 3150802), Bacto agar (BD DF0140-15-4), D-glucose 6-phosphate potassium salt (Sigma-Aldrich; G6526-1G, lot SLBH9805V), and fosfomycin disodium salt (Sigma-Aldrich; P5396, lot 125M4169).

### Base media preparation

#### Mueller–Hinton–poloxamer base

Poloxamer 407 was dissolved in deionized water to a final concentration of 25% (wt/vol) by stirring overnight at 4°C. One-fifth volume of 5× Mueller–Hinton broth (10.5% wt/vol) was then added to yield a final mixture containing 20% Poloxamer 407 and 1× Mueller–Hinton broth. Filter-sterilized D-glucose-6-phosphate potassium salt was added to a final concentration of 25 µg/mL. The solution was autoclaved, cooled, and stored at 4°C in liquid form until use.

#### Agar dilution base

Mueller–Hinton agar was prepared by combining Mueller–Hinton broth with 1.5% (wt/vol) Bacto agar, autoclaving, and maintaining the molten medium at ~50°C in a water bath. Filter-sterilized D-glucose-6-phosphate potassium salt was then added to a final concentration of 25 µg/mL.

#### Agar dilution plates

Doubling dilutions of fosfomycin (512–0.25 µg/mL) were prepared by adding appropriate volumes of filter-sterilized fosfomycin stock to aliquots of agar dilution base medium. Plates (15 mL per plate) were poured, allowed to solidify at room temperature, and used the following day.

#### Poloxamer dilution plates

For poloxamer dilution plates, filter-sterilized fosfomycin stock was added to pre-made Mueller–Hinton poloxamer base aliquots stored at 4°C, gently mixed by swirling, and dispensed into plates (15 mL per plate). Plates were allowed to solidify at room temperature prior to use.

#### Accuracy and precision analysis

Fosfomycin susceptibility testing of all controls and clinical isolates (*n* = 82) was performed in parallel using the poloxamer dilution and agar dilution method reference method. For all clinical isolates examined, susceptibility testing was repeated three times, with each biological replicate performed on a separate day and both methods conducted in parallel. Prior to each day of testing, isolates were re-streaked on non-selective blood agar plates and incubated at 37°C for 18–24 hours. Single colonies from isolates were suspended in sterile Mueller–Hinton broth to 0.5 McFarland using a bioMérieux DensiCHEK Plus instrument. Dilutions of the suspensions (1:10) were loaded into inoculum wells of a sterilized Steers replicator, allowing delivery of 2 µL or 10^4^ CFU per spot onto poloxamer dilution and agar dilution doubling plates in parallel in 35 isolate batches. Plates were incubated at 37°C for 16–20 hours and visually scored to determine the MIC of each strain, defined as the lowest antimicrobial concentration at which bacterial growth was completely inhibited. *E. coli* ATCC 25922 and *E. faecalis* ATCC 29212 were also tested in parallel with each experiment and were within acceptable quality control ranges for both poloxamer dilution and agar dilution for all experiments. Two fosfomycin-resistant, *fosA* AR-Bank *E. coli* isolates and a fosfomycin-susceptible *E. coli* clinical isolate were also included on each day of testing.

Raw growth/no-growth reads for each replicate are provided in Table S2, and replicate-specific MIC determinations derived from these reads are shown in Table S3. For method comparison, a single consensus MIC was assigned to each isolate for each method (Table S1). If at least two of the three replicates agreed, the modal MIC was used. If all replicate MICs differed or only two valid replicates were available (e.g., due to skipped dilutions) and they disagreed, the median doubling dilution (rounded to the nearest dilution when between tested values) was assigned. Skipped dilutions were handled per CLSI M07 guidelines with the MIC read above the highest concentration with observed growth. Isolate readings with more than one consecutive or non-consecutive skipped dilutions were considered invalid. These consensus MICs were then compared between poloxamer dilution and agar dilution to calculate essential agreement (EA, ±1 doubling dilution) and categorical agreement (CA) according to established guidelines (15). For all agreement calculations, agar dilution results were used as the reference standard. Results were also considered in EA if both poloxamer dilution and agar dilution yielded the same off-scale results (i.e., >512). CA was established if both poloxamer dilution and agar dilution yielded the same categorical result. Very major errors (VMEs) were defined as poloxamer dilution susceptible and agar dilution resistant, major errors as poloxamer dilution resistant and agar dilution susceptible, and minor errors as one result intermediate and the other susceptible or resistant. Wilson score confidence intervals were calculated using RStudio.

Precision essential agreement (PEA) analysis for the entire data set was determined by assessing consistency among triplicate MIC determinations for each isolate. PEA was defined as all three replicates yielding MICs within ±1 doubling dilution of each other. Precision categorical agreement (PCA) was defined as all three replicates yielding the same categorical interpretation. If only two biological replicates of the MIC determination were valid, PEA and PCA were scored if both replicates were in agreement. Isolates with only one valid replicate were excluded from precision analyses. For each method, the proportion of isolates demonstrating PEA and PCA was calculated, and proportions were compared between the agar dilution and poloxamer dilution methods using the Exact McNemar’s Test in RStudio. A *P* value of <0.05 was considered statistically significant.

Statistical analysis was performed using the indicated methods in R (R Core Team, Vienna, Austria) in RStudio (Posit Software, PBC, Boston, MA).

## RESULTS

### Verification results

**Fig 1:**
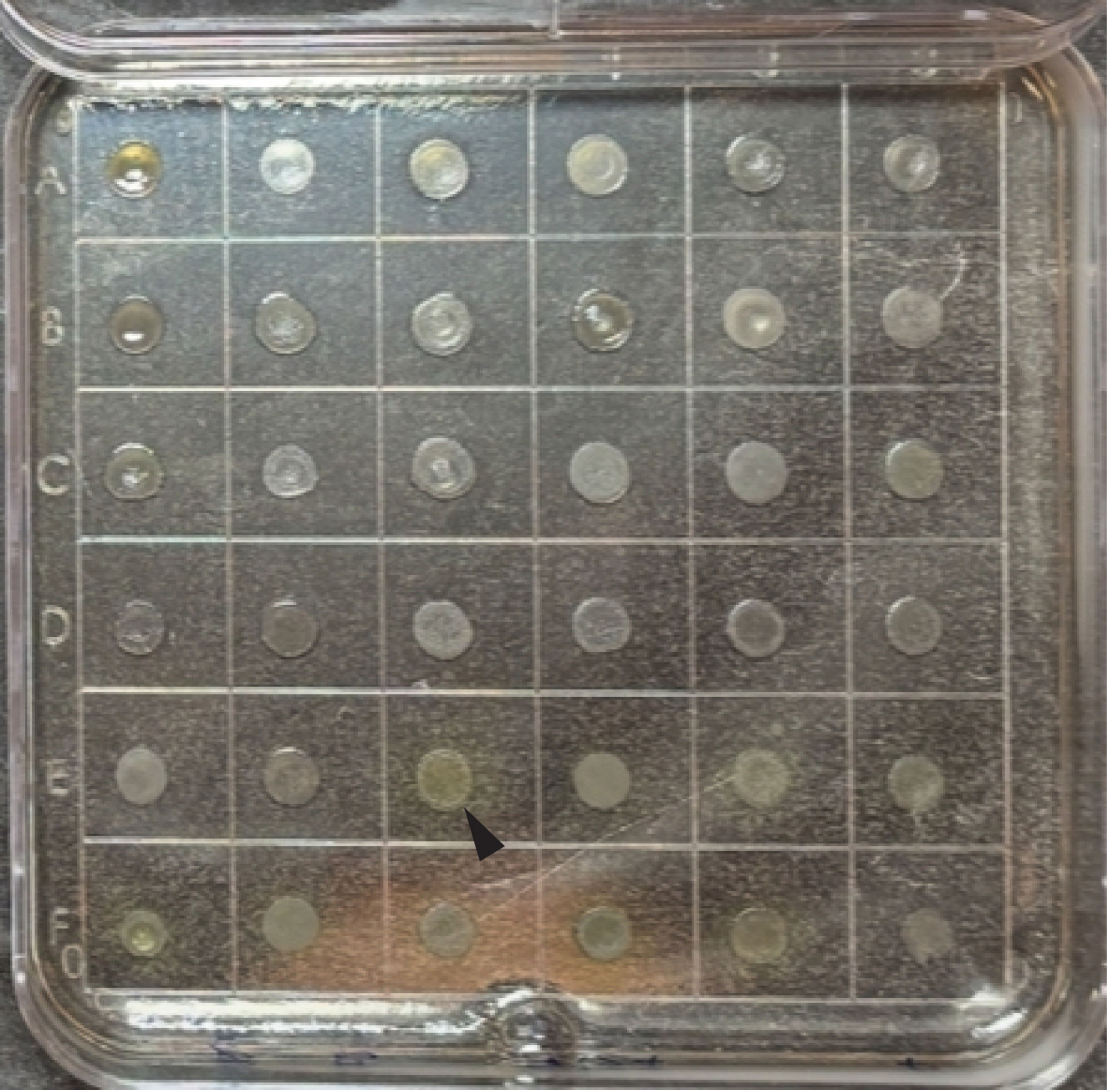
Poloxamer dilution testing example. A representative poloxamer dilution plate inoculated with a Steers replicator is shown. Each spot of contained growth is a unique clinical isolate. Visibly apparent production of pyoverdine in *P. aeruginosa* isolates (arrowhead) can be observed on optically clear poloxamer media.

The accuracy of fosfomycin poloxamer dilution compared with agar dilution was evaluated against 78 isolates of species commonly isolated from urinary tract infections. Categorical and essential agreements are summarized in Table 1. An example of a poloxamer dilution plate is shown in Fig. 1.

**TABLE 1:** Percent accuracy of poloxamer dilution compared with reference agar dilution^*b*^

Species	Total *n*	% CA (95% CI)^*a*^	% VME (95% CI)	% ME (95% CI)	% MinE (95% CI)	% EA (95% CI)
*E. coli*	31	100.0 (89.0–100.0)	0 (0.0–11.0)	0 (0.0–11.0)	0 (0.0–11.0)	87.1 (71.1–94.9)
*Klebsiella* spp.	20	60.0 (38.7–78.1)	0 (0.0–16.1)	10 (2.8–30.1)	30.0 (14.5–51.9)	55.0 (34.2–74.2)
Other *Enterobacterales*	17	82.4 (59.0–93.8)	0 (0.0–18.7)	0 (0.0–18.7)	17.7 (6.2–41.0)	82.4 (59.0–93.8)
*P. aeruginosa*	10	60.0 (31.2–83.1)	0 (0.0–27.8)	0 (0.0–27.8)	40.0 (16.8–68.7)	100.0 (77.2–100.0)

^*a*^ CLSI interpretive criteria for fosfomycin are established only for *E. coli* (and *Enterococcus faecalis*). For *Klebsiella pneumoniae* and other organisms, categorical agreement determinations are based on application of *E. coli* breakpoints to allow method-to-method comparison. No accepted clinical breakpoints exist for these species.

^*b*^ ME, major error; MinE, minor error.

**Fig 2:**
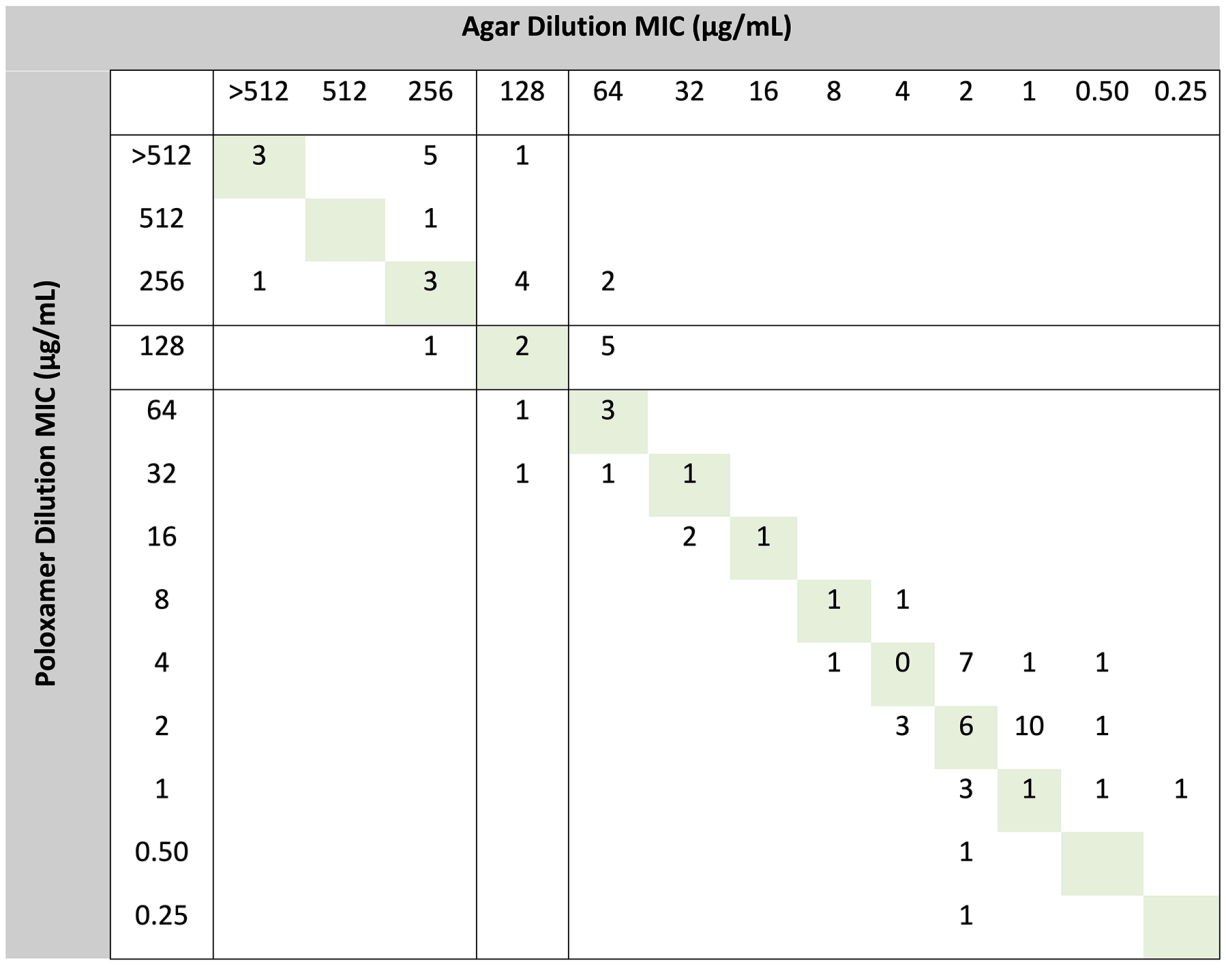
Scatterplot of poloxamer dilution versus agar dilution MIC results. There was a slight positive bias (more conservative MIC call) for poloxamer dilution compared with agar dilution. Per-species breakdown is provided in the supplemental data (see Table S4). *E. coli* isolates ATCC 25922, AR-346, and AR-549 are not shown but were all observed to be in EA and CA.

**Fig 3:**
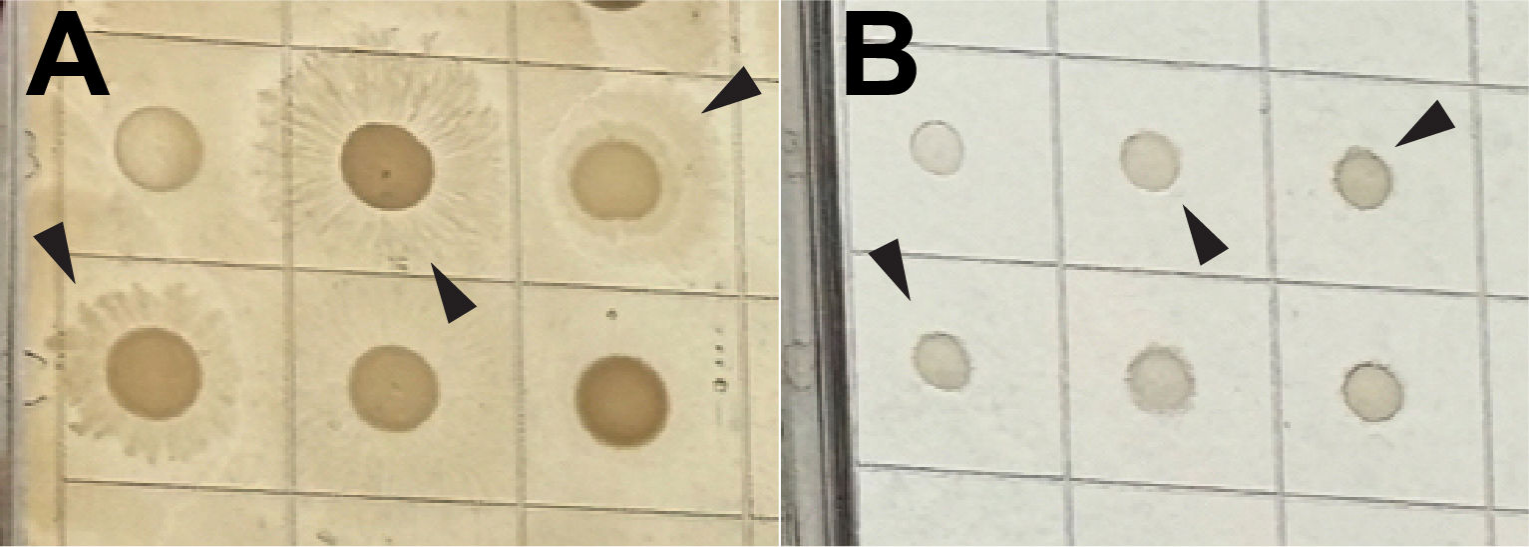
Differential swarming motility of *Proteus* spp*.* on agar and poloxamer. An image of bacterial isolate growth on an agar dilution plate (**A**) and a poloxamer dilution plate (**B**), both containing *Proteus* strains. Concentric swarming isolates of *Proteus* on agar (arrowheads) are not observed on poloxamer dilution plates.

Accuracy was high for *E. coli*, additional *Enterobacterales* species examined, and *P. aeruginosa* (Table 1). No VME were observed. Two major errors were observed for *Klebsiella pneumoniae. E. coli* strains, with the exception of the two pre-selected *fosA* strains from the AR Bank (MIC >512 µg/mL for both methods), were all highly susceptible, and EA and CA were 87% and 100%, respectively (*n* = 31). There was lower CA for *P. aeruginosa* and *Klebsiella* spp., likely reflecting MICs clustering near fosfomycin interpretive criteria extrapolated from *E. coli*. EA was also lower for *Klebsiella* spp. based on several strains which tested at >512 µg/mL for poloxamer dilution and 256 or 128 µg/mL by agar dilution. Overall, there was a bias toward higher and, therefore, more conservative poloxamer dilution MICs compared with agar dilution, demonstrated in the scatterplot for all data (Fig. 2). Individual scatterplots for *E. coli*, *K. pneumoniae*, other *Enterobacterales*, and *P. aeruginosa* are provided in Table S4. Skipped dilutions, as has been previously observed for agar dilution (5), occurred with agar dilution and poloxamer dilution inconsistently among biological replicates (Table S2). For *E. coli*, single and double skipped dilutions were approximately six times more frequent for agar dilution than poloxamer dilution (Table 2; *P <* 0.0001 and *P* = 0.02, respectively; Fisher’s exact test). *Proteus* swarming was completely inhibited on poloxamer dilution at 16–20 hours of incubation, whereas characteristic concentric swarming was observed on agar dilution under the same conditions (Fig. 3).

**TABLE 2:** Percentage of single skipped dilutions observed in testing events for poloxamer versus agar dilution^*a*^

Species	Method	*n*	Single skipped dilutions (%)	*P* value	2+ skipped dilutions (%)	*P* value
*E. coli*	Agar	90	33 (36.7)	0.000	11 (12.2)	0.018
	Poloxamer	90	6 (6.7)		2 (2.2)	
*Klebsiella* spp.	Agar	60	13 (21.7)	0.132	4 (6.7)	0.119
	Poloxamer	60	6 (10.0)		0 (0.0)	
*P. aeruginosa*	Agar	30	8 (26.7)	0.333	4 (13.3)	0.353
	Poloxamer	30	4 (13.3)		1 (3.3)	
*Proteus* spp*.*	Agar	24	1 (4.2)	0.097	0 (0.0)	0.489
	Poloxamer	24	6 (25.0)		2 (8.3)	
*Citrobacter* spp*.*	Agar	12	1 (8.3)	0.069	0 (0.0)	0.478
	Poloxamer	12	6 (50.0)		2 (16.7)	
*E. cloacae* complex	Agar	6	1 (16.7)	1.000	0 (0.0)	1.000
	Poloxamer	6	2 (33.3)		1 (16.7)	
*P. rettgeri*	Agar	3	0 (0.0)	0.400	0 (0.0)	1.000
	Poloxamer	3	2 (66.7)		0 (0.0)	
*Salmonella* spp*.*	Agar	3	0 (0.0)	0.400	0 (0.0)	1.000
	Poloxamer	3	2 (66.7)		0 (0.0)	
*M. morganii*	Agar	3	0 (0.0)	0.100	0 (0.0)	1.000
	Poloxamer	3	3 (100.0)		0 (0.0)	
All species	Agar	231	57 (24.7)	0.028	19 (8.2)	0.046
	Poloxamer	231	37 (16.0)		8 (3.5)	

^*a*^ *n* denotes the number of separate MIC determinations. *P *value comparisons were performed using Fisher's exact test.

### Precision

Precision agreement among the three biological triplicates for each isolate was calculated to compare reproducibility of poloxamer dilution compared with agar dilution. Interestingly, precision essential agreement for poloxamer dilution (74%) was higher than for agar dilution (63%); however, this did not reach statistical significance (*P* = 0.2). The lower PEA for agar dilution was attributable in part to increased variability in measured results caused by skipped dilutions. The PCA for poloxamer dilution (81.6%) was marginally higher than that for agar dilution (76.3%), a difference that likewise did not reach statistical significance (*P* = 0.5, Exact McNemar Test; Table S5).

## DISCUSSION

In this study, we highlight the potential of poloxamer dilution as an accurate and precise, on-demand alternative to agar dilution for antimicrobial susceptibility testing. Using fosfomycin testing as a use case, poloxamer dilution performed well relative to the agar dilution reference method whose performance was compromised by the more frequent occurrence of skipped dilutions (5). For *E. coli*, poloxamer dilution clearly distinguished susceptible from resistant *fosA* strains, and poloxamer dilution also had reasonable essential agreement for *Enterobacterales* and *P. aeruginosa* compared with agar dilution. Errors were almost always conservative across species. *Klebsiella* spp., which constituted approximately 25% of isolates tested, most of which were fosfomycin intermediate or resistant by agar dilution, contributed to approximately 50% of the total categorical and essential agreement errors, all of which were conservative (i.e., reading at a higher MIC by poloxamer dilution). Therefore, demonstration of fosfomycin susceptibility by poloxamer dilution is unlikely to result in falsely susceptible interpretations, while acknowledging that PK/PD data remain insufficient for organisms other than *E. coli* and *E. faecalis*. Notably, *Klebsiella* MICs by both methods were higher than those of other organisms tested and were associated with frequent skipped wells and greater method discordance, suggesting that fosfomycin susceptibility results for this species should be interpreted with caution in the absence of species-specific PK/PD data for urinary tract infection.

Because of its thermoreversible properties, autoclaved poloxamer media can be aliquoted and stored sterile in liquid form at 4°C, allowing immediate setup of poloxamer dilution testing. In contrast to agar dilution, this eliminates the need for repeated media melting, maintenance within a narrow temperature range, and time-sensitive antibiotic incorporation steps. This same property permits antibiotics to be added to cold, liquefied poloxamer without risking heat-associated degradation that can occur during preparation of agar dilution plates.

Poloxamer dilution testing can also be performed in microwell plates (Supplementary Methods; Fig. S1). In previous work, we validated a digital dispensing method using an inkjet printer to rapidly generate custom doubling-dilution antimicrobial panels on demand, with performance comparable to reference dilution methods (6, 10, 15–17). Using this approach, poloxamer dilution can be readily adapted to microwell formats while maintaining high categorical and essential agreement. In a pilot application, microwell poloxamer dilution testing of fosfomycin against 25 *E. coli* clinical isolates demonstrated 96% categorical agreement and 96% evaluable essential agreement compared with CE-marked agar dilution reference plates (Table S6), supporting the suitability of this format.

Taken together, these findings position poloxamer dilution as a practical and reliable method that can be implemented within the local clinical microbiology laboratory, bringing reference-level antimicrobial susceptibility testing capabilities closer to the site of patient care. While agar dilution can be implemented in different ways, depending on batch size and laboratory workflow, poloxamer dilution eliminates the need for repeated agar melting, temperature control, and time-sensitive antibiotic incorporation, thereby simplifying setup and enabling true on-demand testing. By enabling flexible, on-demand preparation of high-quality dilution panels, poloxamer dilution technology has the potential to expand testing portfolios and substantially reduce turnaround time while maintaining the analytical rigor of reference methods. Further studies are warranted to explore the applicability of this approach across a broader range of bacterial species and antimicrobial classes.

## SUPPLEMENTAL MATERIAL

Supplemental materials jcm.01822-25-s0001.pdf Supplemental methods and Figure S1.

Supplemental tables jcm.01822-25-s0002.xlsx Tables S1 to S6.

ASM does not own the copyrights to Supplemental Material that may be linked to, or accessed through, an article. The authors have granted ASM a non-exclusive, world-wide license to publish the Supplemental Material files. Please contact the corresponding author directly for reuse.
