# Supplementary material for: Poloxamer dilution as an on-demand alternative to agar dilution-based antimicrobial susceptibility testing
Source: J Clin Microbiol. 2026 Mar 5;64(4):e01822-25. doi: 10.1128/jcm.01822-25 (PMC13059702; doi:10.1128/jcm.01822-25)

Supplementary Materials and Figure

Poloxamer dilution as an on-demand alternative to agar dilution-based antimicrobial susceptibility testing

Matthew T.J. Uy[1,2], Andrea Kirmaier[1*], Lindsey M. Rudtner[1*], Aidan Pine[1,2], James E. Kirby[1,3#]

[1] Beth Israel Deaconess Medical Center, Boston, MA, USA

[2] Northeastern University, Boston, MA, USA

[3] Harvard Medical School, Boston, MA, USA

*** Equal contributors, listing is in alphabetical order.**

#Address correspondence to: James E. Kirby, jekirby@bidmc.harvard.edu

Supplementary Materials and Methods

Poloxamer dilution testing in 48-well microplate format. As described (1), digital dispensing technology was used to print doubling dilutions of fosfomycin stock solution into a dry 48-well plate. Previously prepared, sterile, liquid Mueller–Hinton–Poloxamer (MH-P) medium containing glucose-6-phosphate was then dispensed into the wells using a Combitip positive-displacement repeat pipettor (Eppendorf AG, Hamburg, Germany). After gentle mixing, plates were allowed to solidify at room temperature. Susceptibility testing was piloted with 25 *E. coli* clinical isolates using CE-marked Liofilchem (Waltham, MA) pre-made 24-well fosfomycin agar dilution plates as the reference comparator. Data are tabulated in Table S6.

Supplemental Reference

1. Smith KP, Kirby JE. 2016. Verification of an Automated, Digital Dispensing Platform for At-Will Broth Microdilution-Based Antimicrobial Susceptibility Testing. J Clin Microbiol 54:2288-93.

Figure S1. Example of poloxamer dilution testing in 48-well microplate format. A microwell format allows testing of a full doubling-dilution range for multiple isolates, including a QC strain, in a single plate.

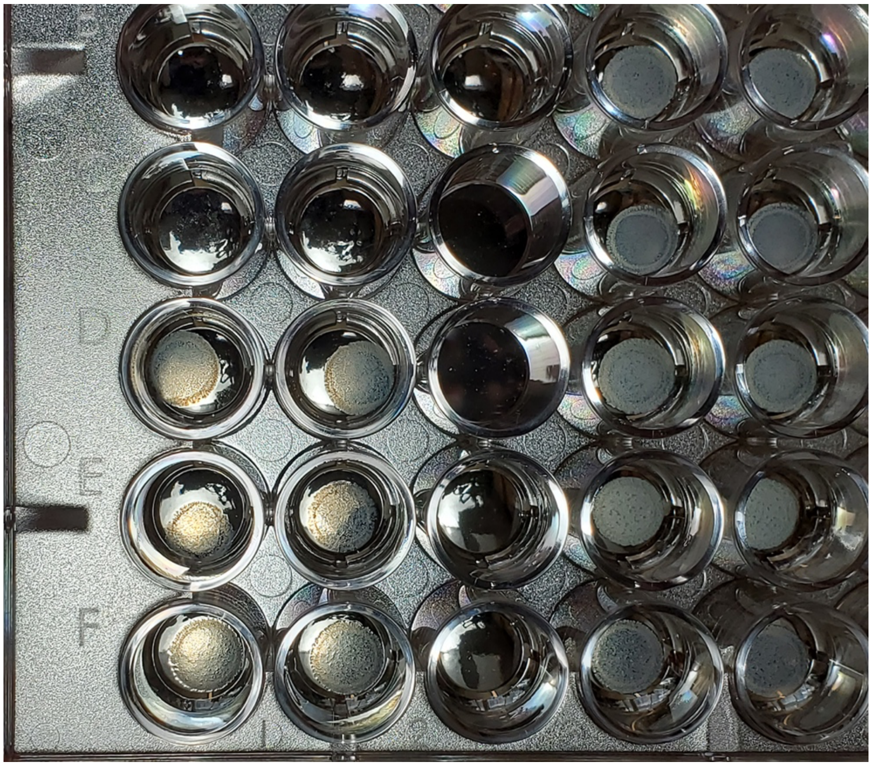

Supplement: Supplemental materials — Supplemental methods and Figure S1. [file jcm.01822-25-s0001.pdf]
